# Efficacy of Ozone against Phosphine Susceptible and Resistant Strains of Four Stored-Product Insect Species

**DOI:** 10.3390/insects8020042

**Published:** 2017-04-11

**Authors:** Xinyi E, Bhadriraju Subramanyam, Beibei Li

**Affiliations:** Department of Grain Science and Industry, Kansas State University, Manhattan, KS 66506, USA; sbhadrir@ksu.edu (B.S.); libeibei@ksu.edu (B.L.)

**Keywords:** fumigation, stored-product insects, phosphine resistance, ozone, mortality, progeny suppression

## Abstract

The efficacy of ozone was evaluated against four economically-important stored-product insect species at 27.2 °C and 20.4% r.h. Adults of phosphine-susceptible laboratory strains and phosphine-resistant field strains of the red flour beetle, *Tribolium castaneum* (Herbst), saw-toothed grain beetle, *Oryzaephilus surinamensis* (Linnaeus), maize weevil, *Sitophilus zeamais* Motschulsky, and rice weevil, *Sitophilus oryzae* (Linnaeus), were exposed in vials to an ozone concentration of 0.42 g/m^3^ (200 ppm) for 1, 2, 3, 5, 6, 8, 10 and 12 h with 0 and 10 g of wheat. Initial and final mortalities were assessed 1 and 5 d after exposure to ozone, respectively. After an 8–12-h exposure to ozone, initial mortality of *Sitophilus* spp. and *O. surinamensis* was 100%, whereas the highest initial mortality of *T. castaneum* was 90%. A 3–4-h exposure to ozone resulted in 100% final mortality of *Sitophilus* spp., whereas *O. surinamensis* required a 6- to 10-h exposure to ozone. Adults of *T. castaneum* were least susceptible to ozone, and after a 10-h exposure, mortality ranged between 82 and 95%. Time for the 5 d 99% mortality (LT_99_) for adults of laboratory and field strains of *Sitophilus* spp., *O. surinamensis* and *T. castaneum* were 2.00–5.56, 4.33–11.18 and 14.35–29.89 h, respectively. The LT_99_ values for adults of *T. castaneum* and *O. surinamensis* were not significantly different between bioassays conducted with 0 and 10 g of wheat. The LT_99_ values for the laboratory strains of *Sitophilus* spp. in the absence of wheat were significantly lower than those obtained in the presence of wheat. Both phosphine-susceptible and -resistant strains were equally susceptible to ozone. Ozone effectively suppressed adult progeny production of all four species. Ozone is a viable alternative fumigant to control phosphine-resistant strains of these four species.

## 1. Introduction

Ozone is a highly reactive gas and has been used as a disinfectant in water treatment plants, as well as in the food-processing industry [1,2]. The decomposition of ozone leads to the formation of free radicals, including O_3_**^•^**^−^, O_2_**^•^**^−^, O**^•^**^−^, **^•^**OH, HO_3_**^•^** and HO_2_**^•^**, which can oxidize sulfhydryl groups found in amino acids, peptides, proteins and unsaturated fatty acids of organisms [1,3,4]. The oxidation of amino acids by ozone is believed to be responsible for the alteration of protein structure and function [3,4]. The half-life of ozone is affected by temperature and relative humidity [5]. At 50% relative humidity (r.h.), the half-life of ozone at 4, 24, 28 and 40 °C is 1850, 850, 650 and 0 min, respectively. In addition to temperature, the moisture content of grain or ambient relative humidity has an impact on the efficacy of ozone against insects and microorganisms [5].

Ozone has been reported to deactivate microflora and degrade mycotoxins and to effectively kill coleopterous and lepidopterous stored-product insect pests [2,6,7,8,9,10]. Due to its highly reactive nature, the rate of ozone penetration through the grain mass is related to the surface characteristics of grain kernels [2,10]. The reaction of ozone within a grain mass has been categorized into two phases: phase I and phase II. During phase I, ozone reacts with the active sites on the kernel surface, and once all sites are saturated, the ozone concentration gradually increases (phase II) to levels lethal for the target insect pests [11,12]. The length of these two phases is affected by the quantity of grain, ozone flow rate, concentration of inlet ozone, grain temperature, and grain moisture content [11,12].

Insects have the ability to evolve resistance to insecticides [13]. Using simulations of population genetic models, researchers found that resistance is more likely to occur if the major genes for resistance to an insecticide are present prior to insecticide exposure [13]. Sousa et al. [14] studied ozone toxicity to the maize weevil, *Sitophilus zeamais* Motschulsky, under selection pressure for two generations and did not find any evidence of resistance development to ozone. Typically, selection pressure should be applied for at least 10 generations to observe any discernable evidence of resistance development.

Widespread resistance in insects associated with stored grain to phosphine [15,16], a commonly-used grain fumigant, necessitates finding alternatives to control phosphine-resistant insects. Ozone is a potential alternative to phosphine; however, its efficacy against phosphine-resistant insects has not been well studied [6]. In this study, both phosphine-susceptible laboratory and -resistant field strains of four economically-important stored-product insect species were exposed to ozone for evaluating its efficacy in controlling phosphine-resistant insects. In addition, the ability of ozone in suppressing adult progeny production of these four species was evaluated.

## 2. Material and Methods

### 2.1. Insect Cultures

Cultures of the red flour beetle, *Tribolium castaneum* (Herbst), were reared on organic wheat flour (Heartland Mills, Marienthal, KS, USA) fortified with 5% (by wt) brewer’s yeast. The rice weevil, *Sitophilus oryzae* (Linnaeus), was reared on organic hard red winter wheat (Heartland Mills). Cultures of *S. zeamais* were reared on organic yellow corn (Heartland Mills) and that of saw-toothed grain beetle, *Oryzaephilus surinamensis* (Linnaeus), on organic rolled oats (Heartland Mills) plus 5% (by wt) brewer’s yeast diet. All cultures were held at 28 °C and 65% r.h. in an environmental growth chamber (Model I-36 VL; Percival Scientific, Perry, IA, USA). Unsexed adults of *T. castaneum* and *O. surinamensis* of mixed ages (1–4 weeks old) were collected directly from culture jars after sifting the culture through an 841-µm opening square-holed sieve. For *Sitophilus* spp., the culture was sifted through a 1.68-mm opening square-holed sieve. Field strains of *T. castaneum* and *O. surinamensis* were collected from farm-stored grain in Kansas, USA, whereas field strains of *S. zeamais* and *S. oryzae* were collected from farm-stored grain in Texas, USA. Phosphine resistance of all species and strains was verified following a discriminating dose test [17], where 50 unsexed mixed-age (1–4 weeks old) adults were exposed to phosphine in triplicate. Phosphine concentrations used during the test for *T. castaneum*, *O. surinamensis* and *Sitophilus* spp. strains were 0.042, 0.052 and 0.042 g/m^3^, respectively (30, 37.5 and 30 ppm; 1 g/m^3^ = 719 ppm at 25 °C). Fumigation lasted for 20 h, and mortality was assessed 14 day after fumigation (Table 1).

### 2.2. Bioassays

Bioassays were carried out in snap cap vials (23 mm in diameter and 55 mm in height) that had mesh bottoms (250 µm opening) and mesh caps with the same size openings to facilitate penetration of ozone through the vials and also to prevent insects from escaping. The ozone exposure was conducted in an air-tight polymethyl methacrylate (PMMA) chamber (0.50 m × 0.35 m × 0.35 m). Ozone was generated by a custom-built corona discharge ozone generator (O_3_Co, Idaho Falls, ID, USA) with a capacity of 2.5 g/h and monitored by a gas analyzer (IN2000-L2-LC, IN USA, Norwood, MA, USA). The ozone concentration in the testing chamber was recorded every minute and acquired by a program written in LABVIEW (National Instruments, Austin, TX, USA). The ozone generator was housed inside a trailer that was parked outside on the North campus of Department of Grain Science and Industry, Kansas State University, Manhattan, KS, USA. The temperature and relative humidity of the chamber were monitored by HOBO^®^ temperature and humidity sensors (Model: U10-003, Onset Computer Corp, MA, USA). The mean ± standard error (SE) (*n* = 266) temperature during tests was 27.2 ± 0.08 °C with the minimum and maximum being 22.9 and 32.5 °C, respectively. The mean ± SE relative humidity during tests was 20.4% ± 0.9%, with the minimum and maximum being 15.0% and 30.6%, respectively. Each vial held 20 adults of a specific species and strain with either 0 or 10 g of wheat. The feeding air flow rate was 0.02 m^3^/minute. Samples were exposed to 0.42 g/m^3^ (200 ppm, 1 g/m^3^ = 476 ppm at 25 °C) of ozone for 1, 2, 3, 4, 5, 6, 8 and 10 h. An additional 12-h exposure was included in bioassays with *O. surinamensis* strains. After the intended exposure to ozone, vials were brought back to the laboratory and kept in the environmental growth chamber at 28 °C and 65% r.h. Prior to incubating in the growth chamber, vials without wheat received 10 g of wheat as food for insects. Mortality was checked 1 and 5 d after ozone exposure. Control vials were kept in the trailer where the ozone exposure was conducted throughout the tests and were handled similarly as the treatment vials. After mortality assessment, dead and live adults were placed back into the vials and held in the growth chamber for 42 d to determine adult progeny production. Vials with *T. castaneum* and *O. surinamensis* received 2 g of organic whole wheat flour and rolled oats, respectively, to provide food for larvae emerging from eggs and to allow normal development to adulthood, because in previous tests, these two species held in vials with only wheat kernels failed to develop to adulthood [18]. Each treatment combination was replicated five times. All bioassays with ozone were conducted between August and October 2015.

### 2.3. Data Analysis

Mortality was expressed as a percentage based on the number of dead insects out of the total exposed. Mortality of exposed insects was corrected for control mortality [19]. The corrected 5 d mortality data were subjected to probit analysis [20] to determine exposure time resulting in 50% (LT_50_) and 99% (LT_99_) mortality of insects. LT_99_ values were compared using ratio tests [21]. If the 95% confidence interval (CI) for the ratio included 1, the difference between the pair being compared was not significant [21]. The number of adult progeny produced was counted, and the initial number of added insects (20) was subtracted from this number. Progeny production by species and strain were transformed to log_10_(x + 1), and subjected to one-way analysis of variance (ANOVA) to determine the effect of exposure time on progeny production for each strain. Means were separated by Bonferroni *t*-tests at *α* = 0.05 [19]. Both the corrected 1- and 5-d mortality were plotted against exposure durations using Sigma Plot^®^ (12.5, SYSTAT 2013, San Jose, CA, USA).

## 3. Results and Discussion

The 5-d mortality of *O. surinamensis* and *Sitophilus* spp. adults after 6–10 and 3–10 h of exposure to ozone was 100%. The 5-d mortality of *T. castaneum* adults was 83%–100% after 8–10 h of exposure to ozone (Table 2). *Sitophilus* spp. generally were more susceptible to ozone than *O. surinamensis* and *T. castaneum*. Other studies also reported *Sitophilus* spp. to be more susceptible to ozone exposure than *T. castaneum*. Kells et al. [11] exposed adults of *T. castaneum* and *S. zeamais* to an ozone concentration of 0.10 g/m^3^ (50 ppm) for 3 d, and the mortality of these two species was 92.2 and 100%, respectively. Hansen et al. [22] exposed adults of *T. castaneum*, *O. surinamensis*, *S. oryzae* and *S. zeamais* to low concentrations of ozone for several days, and complete mortality was observed at the following concentrations for each species: 0.074 g/m^3^ (35 ppm) for 6 d for *T. castaneum*, 0.042 g/m^3^ (20 ppm) for 5 d for *O. surinamensis*, 0.044 g/m^3^ (21 ppm) for 5 d for *S. oryzae* and 0.164 g/m^3^ (78 ppm) for 5 d for *S. zeamais*. *S. zeamais* adults were least susceptible to ozone followed by *T. castaneum*, *O. surinamensis* and *S. oryzae*. McDonough et al. [8] exposed adults of *T. castaneum*, *S. oryzae* and *S. zeamais* to an ozone concentration of 1800 ppm (3.84 g/m^3^), and complete mortality was obtained after 120, 60 and 120 h, respectively.

Ozone can cause oxidative damage to insects, and to deal with this oxidative stress, one of the strategies insects use is to lower the respiration rate by breathing discontinuously [23]. One hundred adults of *S. oryzae* and *T. castaneum* were exposed to 0.2 g/m^3^ ozone for 2 h, and the carbon dioxide production of was 6.0 and 2.6 µl/mg of insects, respectively. However, corresponding carbon dioxide production of these two species in the control was 6.5 and 3.6 µl/mg of insects, respectively [23]. Regardless whether ozone was present or not, adults of *S. oryzae* generated close to one-fold more carbon dioxide than adults of *T. castaneum*, indicating that *S. oryzae* has a higher respiration rate than *T. castaneum* [23]. These differences in respiration rates may have contributed to *Sitophilus* spp. being more susceptible to ozone than *T. castaneum* that we observed in our study.

The probit estimates for lethal times (LT_50_ and LT_99_) are shown in Table 3 and Table 4. Based on 5-d mortality, in the presence of 10 g of wheat, LT_50_ and LT_99_ values for *T. castaneum*, *O. surinamensis*, *S. zeamais* and *S. oryzae* were 2.85–4.99 and 13.64–25.81, 1.16–2.28 and 7.07–11.18, 1.75–1.92 and 3.80–5.56 and 1.32–1.51 and 2.00–3.11 h, respectively. *Sitophilus* spp. tended to be more susceptible to ozone, and LT values were less than those of *O. surinamensis* and *T. castaneum* by 2–5-fold. Bonjour et al. [24] exposed adults of *T. castaneum* and *S. oryzae* to 0.10 g/m^3^ (50 ppm) of ozone for one day inside a steel grain bin, which contained 13,600 kg of hard red winter wheat. Adults of each species were confined in cotton muslin tea bags with 50 g of wheat kernels. The survival of *T. castaneum* and *S. oryzae* was 100 and 35%, respectively, indicating that *T. castaneum* is more tolerant to ozone than *S. oryzae*.

In our study, when insects were exposed to ozone without wheat, LT_50_ and LT_99_ values for *T. castaneum*, *O. surinamensis*, *S. zeamais* and *S. oryzae* were 3.76–4.78 and 10.65–29.89, 0.98–2.10 and 4.33–9.05, 0.80–1.32 and 3.14–4.20 and 0.92–1.10 and 2.32–2.91 h, respectively. As expected, *Sitophilus* spp. were more susceptible to ozone than the other species. Sousa et al. [6] exposed 20 unsexed adults of *T. castaneum* and *O. surinamensis* collected from 23 locations in Brazil to 0.32g/m^3^ (150 ppm) of ozone in the absence of food. The LT_99_ values based on 8-d mortality were 22.17–37.90 h for *T. castaneum* and 11.03–18.72 h for *O. surinamensis*, and these values are comparable to our results.

The effect of adding 10 g wheat on the efficacy of ozone at 0.42 g/m^3^ was not significant (Table 5). Ratio tests showed that there were no significant differences between the 5-d LT_99_ values of samples with 0 and 10 g of wheat, except for *S. zeamais* and *S. oryzae* laboratory strains, where LT_99_ values were higher when wheat was present during ozone exposure. As an oxidizing agent, ozone can react with the active sites on the surface of the wheat kernels prior to targeting insects [11]. However, when a small amount of wheat was present during the exposure, time for ozone to saturate the active sites can be marginal compared to the total exposure time. Insects were exposed to a similar concentration of ozone regardless of whether wheat was present or absent.

Based on the ratio test results, phosphine-susceptible and -resistant strains of the same species had a similar LT_99_ value regardless of the presence or absence of wheat (Table 6). However, for *S*. *zeamais*, in the absence of wheat, the phosphine-susceptible laboratory strain showed higher LT_99_ values than the corresponding phosphine-resistant field strain. The LT_99_ value of the *S. oryzae* Lab strain was lower than the TX strain in the absence of wheat. When wheat was not present during ozone exposure, the *T. castaneum* Lab strain had a significantly higher LT_99_ value than the PD strain; and *O. surinamensis* AB2 strains had a significantly higher LT_99_ value than the Lab strain.

Ozone oxidizes sulfhydryl groups (-SH) of proteins and double bonds of polyunsaturated fatty acids, and as ozone gets degraded to oxygen, reactive oxygen species are generated that could induce oxidative stress to organisms [1]. Destruction of these vital molecules can lead to cellular lesion and death in insects [25]. Holmstrup et al. [25] did not find up regulation of the focal genes they studied after exposing *T. castaneum* adults to ozone for 24 h, but reported down regulation of many genes. With a 24-h exposure to ozone, the adult survival rate slightly decreased after 3 d, but when exposed to ozone for 48 h, the adult survival rate greatly decreased after 3 d. They recommended that more gene expression studies are needed at several temporal scales to reveal when specific focal genes would show a response. Phosphine is known to inhibit aerobic respiration in several organisms [26,27,28,29,30]. Phosphine inhibits cytochrome *c* oxidase in the mitochondrial electron transport chain [26,31]. Other proposed modes of action for phosphine include chemical reaction with hydrogen peroxide to form the hydroxyl radical, which can cause oxidative damage [32]. This finding was supported by in vivo and in vitro studies using mammalian cell lines exposed to phosphine [32,33,34]. Phosphine was shown to inhibit the antioxidant enzymes catalase and peroxidase [35]. Liu et al. [36] using the larvae of the common fruit fly, *Drosophila melanogaster* Meigen, have shown that phosphine reduced the aerobic respiration rate with an increase in hydrogen peroxide and lipid peroxidation. It seems phosphine and ozone target different components of the cells, which may explain the lack of cross-resistance between these two gases.

**Figure 1:**
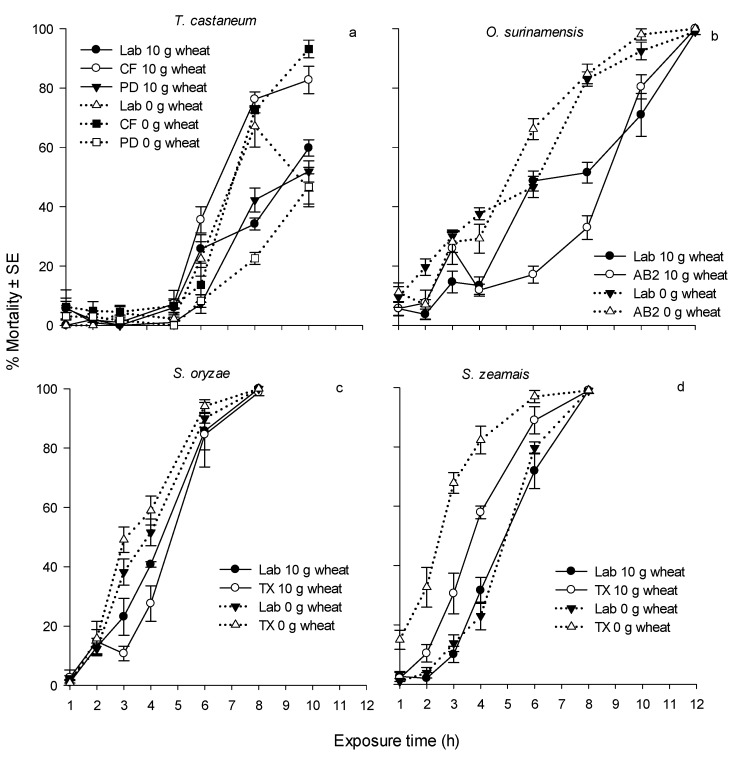
Corrected 1-d mortality of four insect species after exposure to an ozone concentration of 0.42 g/m^3^ for different durations. a. Three strains of *T. castaneum* were exposed to ozone with 0 and 10 g of wheat; b. Two strains of *O. surinamensis* were exposed to ozone with 0 and 10 g of wheat; c. Two strains of *S. oryzae* were exposed to ozone with 0 and 10 g of wheat; d. Two strains of *S. zeamais* were exposed to ozone with 0 and 10 g of wheat. The 1-d mortality in the control treatments with 10 g of wheat for *T. castaneum*, *O. surinamensis*, *S. zeamais* and *S. oryzae* was 0–3%, 2.8%–3.7%, 4% and 0–1.8%, respectively; and the 1-d mortality in the control treatments with 0 g of wheat for *T. castaneum*, *O. surinamensis*, *S. zeamais* and *S. oryzae* was 0–2.9%, 7.0%–13.0%, 1.1%–3.0% and 2%, respectively.

**Figure 2:**
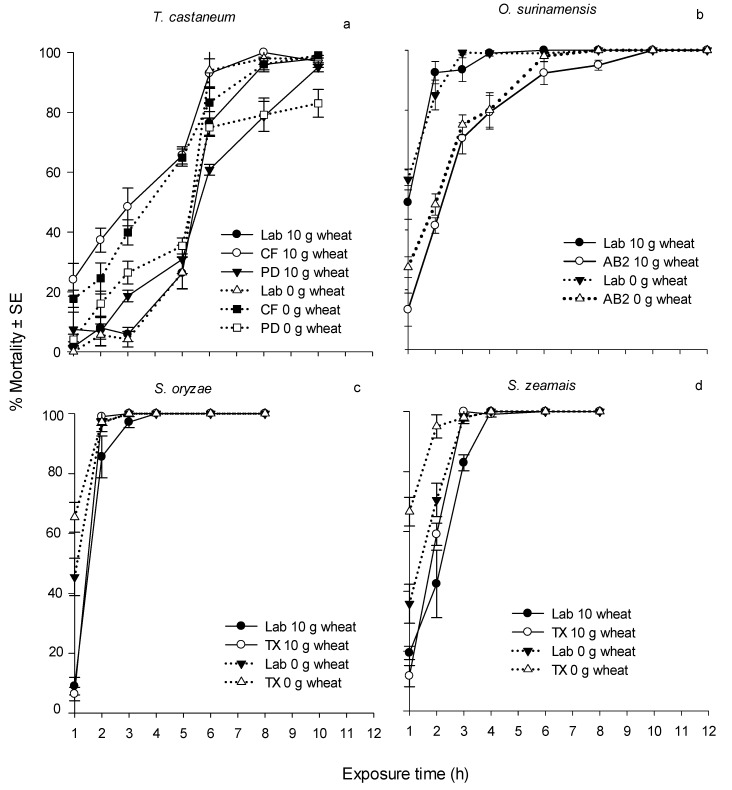
Corrected 5-d mortality of four insect species after exposure to an ozone concentration of 0.42 g/m^3^ for different durations. a. Three strains of *T. castaneum* were exposed to ozone with 0 and 10 g of wheat; b. Two strains of *O. surinamensis* were exposed to ozone with 0 and 10 g of wheat; c. Two strains of *S. oryzae* were exposed to ozone with 0 and 10 g of wheat; d. Two strains of *S. zeamais* were exposed to ozone with 0 and 10 g of wheat. The 1-d mortality in the control treatments with 10 g of wheat for *T. castaneum*, *O. surinamensis*, *S. zeamais* and *S. oryzae* was 0–10.7%, 6.3%–15.2%, 6.0%–7.7% and 2.0%–3.9%, respectively; and the 5-d mortality in the control treatments with 0 g of wheat for *T. castaneum*, *O. surinamensis*, *S. zeamais* and *S. oryzae* was 0.9%–16.3%, 10.6%–12.5%, 3.1%–5.7% and 10.9%–20.5%, respectively.

Progeny was observed in both control and most treatment vials, and results are shown in Table 7 and Table 8. In the presence of wheat, there was significant progeny reduction compared to the control (Table 7) for all tested strains except for the *T. castaneum* PD strain (*F* = 0.35, df = 7, 32, *p* = 0.9256). The *T. castaneum* CF strain showed a relatively low adult progeny in the control treatment (mean ± SE, 14.2 ± 1.0 adults per vial), and the highest progeny reduction was only 25.4% (after exposure to ozone for 6 h). For the rest of the strains and species, the highest progeny reduction ranged from 96.9%–100%. The lack of adult progeny reduction in *T. castaneum* is attributed to the low mortality of adults as a function of ozone exposure time (Figure 1 and Figure 2). The mean ± SE 1- and 5-d mortalities of the *T. castaneum* PD strain exposed to ozone after 10 h with 10 g of wheat were 51.9% ± 3.6% and 95.1% ± 1.5%, respectively. It is reasonable to believe that *T. castaneum* adults can still reproduce after the short exposure to ozone. In addition, ozone does not leave a residue [37,38]. Therefore, eggs and larval stages present after exposure were able to survive and develop into adults.

Greater progeny reduction was observed in *O. surinamensis*, *S. zeamais* and *S. oryzae* strains. In the presence of wheat, the mean control adult progeny production of laboratory and field strains of *O. surinamensis* was 260.6 and 355.8 adults per vial, respectively, and the mean adult progeny in the treated vials of these two strains was 2.4 and 3.8 adults per vial, respectively, after a 4-h ozone exposure (Table 7). The mean adult progeny production of Lab and TX strains of *S. zeamais* in the control treatment was 278.4 and 160.4 adults per vial, respectively. After a 4-h exposure to ozone, adult progeny production of these two strains was 4.8 and 3.8 adults per vial for the Lab and TX strains, respectively. Similarly, without exposure to ozone, the mean control adult progeny production of the Lab and TX strains of *S. oryzae* was 223.0 and 190.8 adults per vial, respectively, and after a 4-h ozone exposure in the presence of wheat, the mean adult progeny was 2.0 and 1.2 adults per vial, respectively. Adult progeny were generally produced by survivors after the ozone exposure. The 1-d mortality of *O. surinamensis* Lab and AB2 strains was 13.4% and 11.9%, respectively, after a 4-h exposure to an ozone concentration of 0.42 g/m^3^, and the mortality increased to 99.0% and 79.3%, respectively, when assessments were made on day 5 (Figure 1 and Figure 2). The increase in mortality over time after brief exposures to ozone suggested delayed toxic effects of ozone in adults of the four species. Delayed toxic effects with ozone have been reported in our earlier studies with adults of the lesser grain borer, *Rhyzopertha dominica* (F.) [39]. The 1-d mortality of the Lab and TX strains of *S. zeamais* was 31.6% and 57.9%, respectively, and that of *S*. *oryzae* was 40.7% and 27.6%, respectively. The 5-d mortality of both species and strains was 100% (Figure 1 and Figure 2). Although *O. surinamensis*, *S. oryzae* and *S. zeamais* strains showed low 1-d mortality at shorter exposure periods to ozone, the corresponding progeny reduction was significant. It is plausible that adults of these three species were able to reproduce less after ozone exposure compared to *T. castaneum*. The discrepancy of progeny reduction among species after ozone exposure was also observed in other studies. After exposure to 1 mg spinosad per kg of wheat, the progeny reduction of *T. castaneum* and *O. surinamensis* strains ranged from 89.8–100% and 68.3%–89.4%, respectively [40]. After exposure to 0.5 mg deltamethrin per kg of wheat, the progeny reduction of *T. castaneum* and *O. surinamensis* strains ranged from 63.3%–100% and 19.1%–100%, respectively [41]. Our previous study demonstrated that exposure of *S. oryzae* and *S. zeamais* in vials with 10 g of wheat to a chlorine dioxide concentration of 0.54 g/m^3^ (200 ppm) for 5 and 10 h, respectively, resulted in 100% progeny reduction. By comparison, to reach 99% progeny reduction, the laboratory and field strains of *R*. *dominica* in the presence of 10 g of wheat needed to be exposed to a chlorine dioxide concentration of 0.54 g/m^3^ for 14.71–15.36 h [18].

Samples without any wheat showed a very similar trend, where progeny reduction was observed after shorter ozone exposures compared to reduction in vials with wheat (Table 8). The *T. castaneum* PD strain again failed to show a significant progeny reduction compared to the control treatment even after a 10-h exposure. The mean ± SE 1- and 5-d mortalities of this strain after a 10-h ozone exposure were 46.7% ± 6.6% and 83.0% ± 4.6%, respectively. The mean ± SE progeny production was 49.4 ± 14.0 adults per vial, which was higher than the control treatment (39.8% ± 4.6% adults per vial). For the other two *T. castaneum* strains, significant adult progeny reduction (94.5%–94.7%) was observed after a 10-h exposure. The progeny reduction of *O. surinamensis* Lab and AB2 strains reached 99.7% after a 4-h ozone exposure. Progeny reduction was 100% for all strains of *Sitophilus* spp. after a two- or four-hour exposure to an ozone concentration of 0.42 g/m^3^.

The reduction in adult progeny production at increasing exposures to ozone can be attributed to an increase in adult mortality. It is also plausible that eggs laid by adults prior to ozone exposure or by adults that survived short exposures of ozone, survived fumigation and successfully developed into F1 adults. This appears to be reasonable, especially in the case of *T. castaneum*. The susceptibility of the eggs to a fumigant is directly related to the architecture of chorion [42]. For stored-product insects, a fumigant can directly enter eggs through respiratory openings (aeropyles) and sperm penetration openings (micropyles) or diffuse through the chorion layers [43,44,45]. However, the rates of gas exchange are expected to be different between these two mechanisms [43,45]. When aeropyles (pseudomicropyles) of the eggs of the hemipteran *Rhodnius prolixus* (Stahl) were sealed with shellac, the oxygen (O_2_) consumption rate was 0.012 mm^3^ O_2_ per egg per h compared to the untreated eggs, which had an O_2_ consumption rate of 0.108 mm^3^ O_2_ per egg per h, indicating that aeropyles facilitate the exchange of gases [43]. The amount and distribution of aeropyles and micropyles vary among eggs of different insect species. Eggs of *T. castaneum* and *O. surinamensis* have a smooth surface and do not have aeropyles or micropyles, suggesting that gas exchange is mainly through diffusion through the chorionic layers [46,47]. The absence of aeropyles and micropyles may have led to less than lethal up-take of ozone during exposures by *T. castaneum* and *O. surinamensis* eggs compared to other species. Therefore, it is possible for these eggs to survive to adulthood. The adults of *T. castaneum* and *O. surinamensis* that emerged from such eggs contributed to the high adult progeny production observed in ozone treatments. *Enoplopactus lizeri* (Hustache) and *Naupactus rugosus* (Hustache), species belonging to the Curculionidae family (weevils), have a few scattered aeropyles on the chorionic surface of the eggs [48]. It is reasonable to assume that the eggs of *Sitophilus* spp., which belong to the Curculionidae family, may have aeropyles like other members in the family. The presence of aeropyles can facilitate the up-take of ozone and result in higher egg mortality [47], resulting in fewer adults emerging in ozone treatments. The smaller number of F1 adult progeny produced by *Sitophilus* spp. exposed to ozone treatments could be due to fewer surviving adults available in these treatments. Examination of Figure 1 and Figure 2, which show increasing mortality of adults, and Table 7 and Table 8, which show a reduction in progeny production, as a function of ozone exposure time, supports this view.

## 4. Conclusions

An ozone concentration of 0.42 g/m^3^ effectively killed adults of phosphine-susceptible and -resistant strains of *T. castaneum*, *O. surinamensis*, *S. oryzae* and *S. zeamais*. In addition, phosphine-susceptible and -resistant strains of each species were equally susceptible to ozone. At this concentration, fumigation for 10 h can completely suppress adult progeny production of *Sitophilus* spp. and *O. surinamensis*, but not that of *T. castaneum* strains. The 10 g of wheat in vials did not significantly affect the efficacy of ozone against the four insect species when compared to those exposed to ozone without wheat. The laboratory findings should be confirmed by tests under practical field conditions, such as grain bins or in grain-processing facilities.

## Figures and Tables

**Table 1:** Sites and year of collection of field strains of four insect species and their susceptibility to discriminating doses of phosphine.

Species	County, State	Commodity	Strain ^a^	Collection Year	Survival (%) ^b^
*T. castaneum*	Russell, Kansas	Wheat	PD	2011	45.0
	Washington, Kanas	Wheat	CF	2011	15.0
*O. surinamensis*	Dickinson, Kansas	Wheat	AB2	2011	1.3
*S. zeamais*	Texas ^c^	Corn	TX	2011	6.7
*S. oryzae*	Texas ^c^	Corn	TX	2011	9.3

^a^ Strain refers to the location where insects were collected. PD, Paradise; CF, Clifton; AB2, Abilene; TX, Texas; ^b^ Phosphine resistance evaluations on these strains were conducted for the first time in July 2015; ^c^ County unknown.

**Table 2:** Exposure times required to achieve complete mortality (100%) after 5 d in adults of the four insect species exposed to an ozone concentration of 0.42 g/m^3^ in vials with 0 and 10 g of wheat.

Species	Strain ^a^	0 g Wheat (h)	10 g Wheat (h)
*T. castaneum*	Lab ^b^	8	8
	CF ^c^	8	10
	PD ^d^	10	10
*O. surinamensis*	Lab	6	8
	AB2	10	8
*S. zeamais*	Lab	4	4
	TX	3	4
*S. oryzae*	Lab	4	3
	TX	3	3

^a^ For strain, see footnote to Table 1; ^b^ The mean ± SE mortality of *T. castaneum* Lab strain with 0 and 10 g of wheat after 8 h of fumigation was 98.0% ± 2.0% and 96.0% ± 2.4%, respectively; ^c^ The mean ± SE mortality of *T. castaneum* CF strain with 0 and 10 g of wheat after 10 h of fumigation was 99.1% ± 0.9% and 97.0% ± 2.0%, respectively; ^d^ The mean ± SE mortality of *T. castaneum* PD strain with 0 and 10 g of wheat after 10 h of fumigation was 83.0% ± 4.6% and 95.1% ± 1.5%, respectively.

**Table 3:** Lethal time (LT_50_ and LT_99_) estimates for adults of four insect species based on 5-d mortality data after exposure to an ozone concentration of 0.42 g/m^3^ in vials with 10 g of wheat.

Species	Strain ^a^	*N* ^b^	Mean ± SE		Lethal Time (h, 95% CI)		χ^2^ (df) ^c^
			Intercept	Slope	LT_50_	LT_99_	
*T. castaneum*	Lab	700	−3.56 ± 1.22	5.18 ± 1.66	4.86 (3.17–6.98)	13.64 (8.64–92.79)	4189.55 (33)
	CF	700	−1.42 ± 0.20	3.11 ± 0.33	2.85 (2.42–3.29)	15.91 (11.79–24.91)	447.45 (33)
	PD	700	−2.28 ± 0.40	3.26 ± 0.55	4.99 (4.05–6.23)	25.81 (16.19–64.99)	1019.84 (33)
*O. surinamensis*	Lab	180	−0.19 ± 0.16	2.97 ± 0.58	1.16 (0.83–1.43)	7.07 (4.24–25.46)	72.79 (9)
	AB2	760	−1.21 ± 0.14	3.37 ± 0.25	2.28 (2.03–2.51)	11.18 (9.33–14.16)	195.84 (36)
*S. zeamais*	Lab	600	−1.42 ± 0.20	5.03 ± 0.49	1.92 (1.72–2.11)	5.56 (4.65–7.20)	250.33 (28)
	TX	560	−1.68 ± 0.22	6.91 ± 0.65	1.75 (1.61–1.88)	3.80 (3.37–4.50)	135.55 (26)
*S. oryzae*	Lab	580	−1.34 ± 0.20	7.45 ± 0.69	1.51 (1.39–1.63)	3.11 (2.76–3.65)	149.12 (27)
	TX	580	−1.55 ± 0.10	12.85 ± 0.64	1.32 (1.28–1.36)	2.00 (1.90–2.13)	29.78 (27)

^a^ For strain, see footnote to Table 1; ^b^ *N* = Total number of insects used in generating the probit regression estimates; ^c^ All χ^2^ values for the goodness-of-fit of the model to the data were significant (*p* < 0.0001), indicating the poor fit of the model to the data.

**Table 4:** Lethal time (LT_50_ and LT_99_) estimates for adults of four species based on 5-d mortality data after exposure to an ozone concentration of 0.42 g/m^3^ in vials with 0 g of wheat.

Species	Strain ^a^	*N* ^b^	Mean ± SE		Lethal Time (h, 95% CI)		χ^2^ (df) ^c^
			Intercept	Slope	LT_50_	LT_99_	
*T. castaneum*	Lab	480	−4.54 ± 1.15	6.69 ± 1.52	4.78 (3.77–5.59)	10.65 (8.31–19.90)	868.37 (22)
	CF	620	−2.30 ± 0.29	4.00 ± 0.42	3.76 (3.31–4.21)	14.35 (11.37–20.30)	341.71 (29)
	PD	660	−1.94 ± 0.20	2.89 ± 0.28	4.70 (4.18–5.27)	29.89 (21.74–47.59)	250.42 (31)
*O. surinamensis*	Lab	800	0.03 ± 0.13	3.61 ± 0.41	0.98 (0.80–1.14)	4.33 (3.49–5.98)	233.45 (38)
	AB2	800	−1.18 ± 0.13	3.67 ± 0.25	2.10 (1.90–2.30)	9.05 (7.64–11.25)	208.33 (38)
*S. zeamais*	Lab	600	−0.55 ± 0.13	4.62 ± 0.42	1.32 (1.18–1.44)	4.20 (3.56–5.29)	147.16 (28)
	TX	600	0.38 ± 0.14	3.92 ± 0.57	0.80 (0.61–0.95)	3.14 (2.52–4.59)	162.42 (28)
*S. oryzae*	Lab	600	−0.29 ± 0.12	7.14 ± 0.78	1.10 (1.02–1.17)	2.32 (2.02–2.87)	116.64 (28)
	TX	160	0.16 ± 0.13	4.67 ± 0.99	0.92 (0.68–1.07)	2.91 (2.07–8.17)	32.13 (6)

^a^ For strain, see footnote to Table 1; ^b^ *N* = Total number of insects used in generating the probit regression estimates; ^c^ All χ^2^ values for the goodness-of-fit of the model to the data were significant (*p* < 0.0001), indicating the poor fit of the model to the data.

**Table 5:** Comparison of LT_99_ values between 0 and 10 g of wheat based on 5-d mortality data.

Species	Strain ^a^	Ratio (95% CI) ^b^
*T. castaneum*	Lab	1.26 (0.58–2.73)
	CF	1.11 (0.71–1.73)
	PD	1.16 (0.57–2.36)
*O. surinamensis*	Lab	1.63 (0.82–3.25)
	AB2	1.24 (0.94–1.63)
*S. zeamais*	Lab	1.32 (1.00–1.75) ^*^
	TX	1.21 (0.89–1.64)
*S. oryzae*	Lab	1.34 (1.08–1.65) ^*^
	TX	1.45 (0.96–2.20)

^a^ For strain, see footnote to Table 1; ^b^ Vials with 10 g of wheat had higher LT_99_ values; ^*^ Significant (*p* < 0.05).

**Table 6:** Comparison of LT_99_ values between phosphine-susceptible and -resistant strains based on 5-d mortality data.

Species	Strains ^a^	Ratio (95% CI)	
		0 g of Wheat	10 g of Wheat
*T. castaneum*	Lab vs. CF	1.18 (0.54–2.58)	1.34 (0.87–2.05)
	Lab vs. PD	1.91 (0.76–4.83)	2.79 (1.70–4.58) ^*^
*O. surinamensis*	AB2 vs. Lab	1.58 (0.81–3.09)	2.09 (1.53–2.87) ^*^
*S. zeamais*	Lab vs. TX	1.46 (1.14–1.87) ^*^	1.34 (0.96–1.86)
*S. oryzae*	Lab vs. TX ^b^	1.55 (1.34–1.79) ^*^	1.25 (0.80–1.95)

^a^ For strain, see footnote to Table 1; Strain mentioned first had a higher LT_99_ value; ^b^ With 10 g of wheat, the Lab strain had a higher LT_99_ value than the TX strain, but with 0 g of wheat, the Lab strain had a lower LT_99_ value than the TX strain; ^*^ significant (*p* < 0.05).

**Table 7:** Adult progeny production (% reduction relative to control treatment) of laboratory and field strains of four insect species after exposure for various durations to an ozone concentration of 0.42 g/m^3^ in vials with 10 g of wheat ^a, b^.

Hours	*T. castaneum*			*O. surinamensis*		*S. zeamais*		*S. oryzae*	
	Lab	CF	PD ^c^	Lab	AB2	Lab	TX	Lab	TX
0	115.8 ± 9.6a	14.2 ± 1ab	41 ± 7.9	260.6 ± 32.2a	355.8 ± 19.8a	278.4 ± 16.1a	160.4 ± 9.9a	223 ± 30.1a	190.8 ± 31.9a
1	70.8 ± 23.1a	16.4 ± 2.9ab	57 ± 20.8	242.4 ± 48.4a	229.4 ± 23.9ab	215.4 ± 17.5a	93.2 ± 10.9a	102.4 ± 20.5a	98.2 ± 36.7a
	(38.9%)	(−15.5%)	(−39.0%)	(7.0%)	(35.5%)	(22.6%)	(41.9%)	(54.1%)	(48.5%)
2	58 ± 11.1a	13.8 ± 3.2ab	41.4 ± 6.9	49.4 ± 21.1c	149.8 ± 7.5ab	125.6 ± 24.9a	56.6 ± 16.4a	4.4 ± 3.1b	1.8 ± 0.9b
	(49.9%)	(2.8%)	(−1.0%)	(81.0%)	(57.9%)	(54.9%)	(64.7%)	(98.0%)	(99.1%)
3	45 ± 11.1a	46.4 ± 6.5a	35.8 ± 8.9	69.4 ± 20.5b	92 ± 23.5b	103 ± 12a	4.0 ± 2.3b	1.4 ± 0.5b	0.6 ± 0.7b
	(61.1%)	(−226.8%)	(12.7%)	(73.4%)	(74.1%)	(63.0%)	(97.5%)	(99.4%)	(99.7%)
4 (5) ^d^	65.2 ± 19.8a	54.4 ± 10.1a	66 ± 15.3	2.4 ± 1.4cd	3.8 ± 1.2c	4.8 ± 2.8b	3.8 ± 2.1b	2.0 ± 0.9b	1.2 ± 0.6b
	(43.7%)	(−283.1%)	(−61.0%)	(99.1%)	(98.9%)	(98.3%)	(97.6%)	(99.1%)	(99.4%)
6	5.6 ± 3.4b	10.6 ± 6.7b	60.6 ± 15.9	2.4 ± 1.2cd	1.8 ± 0.6c	0 ± 0.3c	1.2 ± 0.5b	1.4 ± 0.5b	4.2 ± 5.0b
	(95.2%)	(25.4%)	(−47.8%)	(99.1%)	(99.5%)	(100%)	(99.3%)	(99.4%)	(97.8%)
8	8.4 ± 8.4b	29.2 ± 11.9ab	46.4 ± 12.6	2.6 ± 0.9cd	0.4 ± 0.5c	1.0 ± 0.3bc	0.2 ± 0.4b	3.8 ± 1.2b	0.4 ± 0.2b
	(92.7%)	(−105.6%)	(−13.2%)	(99.0%)	(99.9%)	(99.6%)	(99.9%)	(98.3%)	(99.8%)
10	3.6 ± 3.6b	19.8 ± 17.6ab	40.6 ± 10.8	1.2 ± 0.9cd	0 ± 0c	2.4 ± 1.2b	0.2 ± 0.2b	0.8 ± 0.7b	0 ± 0b
	(96.9%)	(−39.4%)	(1.0%)	(99.5%)	(100%)	(98.9%)	(99.9%)	(99.6%)	(100.1%)
12	- ^e^	- ^e^	- ^e^	0.4 ± 0.8d	0.6 ± 0.4c	- ^e^	- ^e^	- ^e^	- ^e^
				(99.8%)	(99.8%)				
*F*-value	12.09	3.38	0.35	17.26	81.73	123.24	21.93	38.90	21.31
df	7, 32	7, 32	7, 32	8, 36	8, 36	7, 32	7, 32	7, 32	7, 32
*p*-value	<0.0001	0.0081	0.9256	<0.0001	<0.0001	<0.0001	<0.0001	<0.0001	<0.0001

^a^ For strain, see footnote to Table 1; ^b^ Means by strains followed by different letters are significantly different (*p* < 0.05, by the Bonferroni *t*-tests); ^c^ There was no significant difference in progeny produced in the control and ozone treatments (*p* > 0.05); ^d^ The exposure time for *T. castaneum* was 5 h and for the rest of the species was 4 h; ^e^ Samples were not exposed for 12 h.

**Table 8:** Adult progeny production (% reduction relative to control treatment) of laboratory and field strains of four insect species after exposure for various durations to an ozone concentration of 0.42 g/m^3^ in vials with 0 g of wheat ^a, b^

Hours	*T. castaneum*			*O. surinamensis*		*S. zeamais*		*S. oryzae*	
	Lab	CF	PD ^c^	Lab	AB2	Lab	TX	Lab	TX
0	47.6 ± 26.8abc	11.4 ± 3.7abc	39.8 ± 4.6	339.6 ± 17.7a	194.8 ± 23a	285.8 ± 11a	179.6 ± 29.3a	162.8 ± 22.8a	114.6 ± 12.8a
1	73.6 ± 17.2a	16.2 ± 2.4abc	65 ± 13.4	282.2 ± 52a	271.6 ± 18.1a	47 ± 23.1b	7.8 ± 3.6b	65.4 ± 9.3b	11.8 ± 6.7b
	(−54.6%)	(−42.1%)	(−63.3%)	(16.9%)	(−39.4%)	(83.6%)	(95.7%)	(59.8%)	(89.7%)
2	46.6 ± 12.9ab	24.4 ± 9.2abc	89.2 ± 13.6	138.2 ± 57.1ab	129.6 ± 23.1a	49.6 ± 28.4b	0.8 ± 0.2bc	0 ± 0c	0 ± 0c
	(2.1%)	(−114%)	(−124.1%)	(59.3%)	(33.5%)	(82.6%)	(99.6%)	(100%)	(100%)
3	46.2 ± 8.4a	69.6 ± 6.7a	92.4 ± 15.5	29 ± 25.8bc	42.6 ± 16.3b	15.4 ± 14.9b	0 ± 0c	0 ± 0c	0 ± 0c
	(2.9%)	(–510.5%)	(–132.2%)	(91.5%)	(78.1%)	(94.6%)	(100%)	(100%)	(100%)
4 (5) ^d^	45.8 ± 14.1a	55.6 ± 19.8ab	95.6 ± 11.3	1 ± 0.7c	0.6 ± 0.7c	0 ± 0b	0.4 ± 0.2c	0 ± 0c	0 ± 0c
	(3.8%)	(–387.7%)	(–140.2%)	(99.7%)	(99.7%)	(100%)	(99.8%)	(100%)	(100%)
6	11.2 ± 11.2bc	46.8 ± 15.9ab	90.8 ± 17.8	0.6 ± 0.7c	0.8 ± 0.6c	0.2 ± 0.4b	0.4 ± 0.4c	0 ± 0c	0 ± 0c
	(76.5%)	(–310.5%)	(–128.1%)	(99.8%)	(99.6%)	(99.9%)	(99.8%)	(100%)	(100%)
8	6 ± 6c	12.6 ± 7.7bc	86 ± 19.8	1.6 ± 0.9c	0.4 ± 0.5c	0 ± 0.3b	0.4 ± 0.2c	0 ± 0c	0 ± 0c
	(87.4%)	(-10.5%)	(–116.1%)	(99.5%)	(99.8%)	(100%)	(99.8%)	(100%)	(100%)
10	2.6 ± 2.6c	0.6 ± 0.5c	49.4 ± 14	0.4 ± 0.5c	0.4 ± 0.5c	0 ± 0c	0 ± 0c	0 ± 0c	0 ± 0c
	(94.5%)	(94.7%)	(–24.1%)	(99.9%)	(99.8%)	(100%)	(100%)	(100%)	(100%)
12	- ^e^	- ^e^	- ^e^	0.6 ± 0.7c	0.4 ± 0.5c	- ^e^	- ^e^	- ^e^	- ^e^
				(99.8%)	(99.8%)				
*F*-value	7.11	4.34	1.92	25.28	59.96	12.36	50.55	1034.93	45.86
df	7, 32	7, 32	7, 32	8, 36	8, 36	7, 32	7, 32	7, 32	7, 32
*p*-value	<0.0001	0.0018	0.0987	<0.0001	<0.0001	<0.0001	<0.0001	<0.0001	<0.0001

^a^ For strain, see footnote to Table 1; ^b^ Means by strains followed by different letters are significantly different (*p* < 0.05, by the Bonferroni *t*-tests); ^c^ There was no significant difference in progeny produced in the control and ozone treatments (*p* > 0.05); ^d^ The exposure time for *T. castaneum* was 5 h and for the rest of the species was 4 h; ^e^ Samples were not exposed for 12 h.
